# State-of-the-Art Software-Based Remote Attestation: Opportunities and Open Issues for Internet of Things

**DOI:** 10.3390/s21051598

**Published:** 2021-02-25

**Authors:** Sigurd Frej Joel Jørgensen Ankergård, Edlira Dushku, Nicola Dragoni

**Affiliations:** DTU Compute, Technical University of Denmark (DTU), 2800 Kgs. Lyngby, Denmark; s164443@student.dtu.dk (S.F.J.J.A.); ndra@dtu.dk (N.D.)

**Keywords:** remote attestation, software-based attestation, timing-based attestation, software integrity verification, legacy Internet of Things

## Abstract

The Internet of Things (IoT) ecosystem comprises billions of heterogeneous Internet-connected devices which are revolutionizing many domains, such as healthcare, transportation, smart cities, to mention only a few. Along with the unprecedented new opportunities, the IoT revolution is creating an enormous attack surface for potential sophisticated cyber attacks. In this context, Remote Attestation (RA) has gained wide interest as an important security technique to remotely detect adversarial presence and assure the legitimate state of an IoT device. While many RA approaches proposed in the literature make different assumptions regarding the architecture of IoT devices and adversary capabilities, most typical RA schemes rely on minimal Root of Trust by leveraging hardware that guarantees code and memory isolation. However, the presence of a specialized hardware is not always a realistic assumption, for instance, in the context of legacy IoT devices and resource-constrained IoT devices. In this paper, we survey and analyze existing software-based RA schemes (i.e., RA schemes not relying on specialized hardware components) through the lens of IoT. In particular, we provide a comprehensive overview of their design characteristics and security capabilities, analyzing their advantages and disadvantages. Finally, we discuss the opportunities that these RA schemes bring in attesting legacy and resource-constrained IoT devices, along with open research issues.

## 1. Introduction

With the Internet of Things (IoT) revolution, IoT devices are experiencing an exponential growth, becoming pervasive in infrastructure and industrial systems (e.g., digital transportation, smart cities, automated factories), and emerging as an integral part of our everyday life (e.g., smart home, wearable devices). According to Statista (https://www.statista.com/statistics/976313/global-iot-market-size/ (accessed on 31 December 2020)), the global IoT market is expected to reach around 1.6 trillion dollars in market revenue by 2025. However, the enormous expansion of interconnected IoT devices that perform safety-critical operations and contain sensitive information, combined with their limited capabilities to implement advanced security techniques, makes IoT devices a prominent target of a broad range of malicious exploitations [1,2,3].

Aimed at securing IoT devices, Remote Attestation (RA) has been proposed as a valuable security technique that allows a trusted party (i.e., verifier) to assure the integrity of the untrusted IoT device (i.e., prover). During the attestation, the prover sends proofs about its current state of the memory (typically a hash of the memory) to the verifier, whereas the verifier matches the received evidence with the expected legitimate state (known in advance) of the prover, and according to that it validates whether the prover is trustworthy or not.

Based on their architectural design, RA schemes can broadly be classified into three main categories: (1) Software-based RA (e.g., Seshadri et al. [4,5]) which provides security guarantees based on strict running time constraints of the verification procedure; (2) Hardware-based RA (e.g., Sailer et al. [6], Tan et al. [7]) which uses a tamper-resistant hardware module as a secure execution environment; and (3) Hybrid RA (e.g., Eldefrawy et al. [8], Brasser et al. [9]) which rely on a minimal read-only hardware-protected memory. Due to the lack of requirements for a specialized tampered-resistance hardware, software-based RA schemes are low-cost solutions in comparison with hardware-based RA. However, using a secure execution environment such as Trusted Platform Module (TPM) [10], ARM TrustZone [11], and Intel Software Guard Extensions (SGX) (https://software.intel.com/en-us/sgx (accessed on 31 December 2020)), hardware-based RA provides high-security guarantees, that protects RA protocol execution from compromised software. Nevertheless, classic low-cost IoT devices do not support the requirements of hardware-based schemes for costly specialized hardware-protected modules. To ensure uninterrupted, safe and secure code execution of the RA protocol, hybrid RA schemes depend on the existence of a minimal read-only hardware-protected memory. However, the assumption made by hardware-based RA and hybrid RA of a specialized hardware is not a trivial requirement for many IoT devices with limited computational power which do not support any specialized hardware, such as battery-free, energy harvesting IoT devices [12].

Considering that there is a great number of legacy IoT devices already deployed without a specialized hardware support, it is difficult (if not impractical) to customize the hardware and redeploy these devices. Due to the cost, it is also not a viable option to replace them all with new devices relying on specialized hardware. In addition, many IoT devices are designed to be small, cheap, and battery-free, thus, introducing new and specialized hardware could potentially not only increase the cost and size of the devices but also deviate from the energy harvesting feature of their design. Nevertheless, it is crucial to provide security protections on such low-cost devices. In this context, software-based RA can be considered a very promising approach. However, to the best of our knowledge, a comprehensive analysis of existing software-based RA schemes in order to investigate their advantages and disadvantages along with the opportunities that they offer for attesting legacy and/or resource-constrained IoT systems is still missing in the literature.

### 1.1. Contributions of the Paper

This paper aims at addressing the aforementioned problem by presenting a comprehensive state-of-the-art of software-based RA schemes in the context of IoT. The overall goal is to provide the reader with the current state-of-the-art of software-based RA protocols, discussing the research opportunities that these schemes bring in attesting legacy and/or resource-constrained IoT devices, along with the research challenges that we still need to address in order to fully adopt these schemes for IoT security. In particular, the paper provides the following main contributions:

- Thorough analysis of the state-of-the-art software-based RA schemes.
- Discussion on opportunities that software-based RA schemes bring in attesting legacy IoT devices, as well as in attesting new emerging IoT devices which rely on Fog computing paradigm and energy harvesting technology.
- Analysis of limitations and research challenges in implementing software-based RA approach.

### 1.2. Paper Outline

The rest of this paper is structured as follows. In Section 2, we briefly summarize related work. Section 3 describes the research methodology used for the literature collection. In Section 4, we give a short background regarding the common system and adversarial model for software-based RA protocols. In Section 5, we present state-of-the-art for RA schemes including their design features, security capabilities, and an analysis of their advantages and disadvantages. In Section 6, we discuss the opportunities of software-based RA scheme and present some open research problems in Section 7. Finally, we provide some concluding remarks in Section 8.

## 2. Related Work

This section summarizes the related works in the RA domain and compares this paper with other RA surveys presented in the literature.

### 2.1. Single Device RA

During the last two decades, many RA approaches have been proposed in the literature. These RA schemes provide different perspectives in terms of architectural design, scalability and security objectives. Based on the architectural design, RA schemes are broadly classified into three main categories: software-based, hardware-based and hybrid-based. Software-based RA schemes, such as Reflection [13], SWATT [4], Pioneer [5], target low-end devices with low-cost design, small size, and low power consumption, which do not provide any hardware support or hardware modification. To establish Root-of-Trust on such devices, software-based approaches rely on highly optimized protocol implementation and make certain adversarial assumptions. To address limited security protections of software-based RA schemes, hardware-based approaches rely on trusted computing architectures such as TPM [10], ARM TrustZone [11], Sancus [14]. Despite their strong security guarantees, the requirement for costly customized hardware that cannot be accommodated in small IoT platforms makes hardware-based protocols incompatible with many low-end devices. To this end, hybrid-based solutions, such as SMART [8], TyTAN [9], TrustLite [15], leverage the best properties of software-based and hardware-based RA approaches to establish Root-of-Trust by relying on minimal hardware assumptions. In particular, hybrid solutions require modification of devices hardware to ensure atomic and secure code execution of RA protocols. One recent work that aims to fill the gap between software-based and hybrid-based RA schemes is SIMPLE [16], a hypervisor-based RA scheme for resource-constrained IoT devices. SIMPLE relies on a software-based memory isolation technique, called Security MicroVisor (SμV) [17]. SμV shields a software-based Trusted Computing Module (TCM) from untrusted application software using selective software virtualisation and assembly-level code verification. However, due to the runtime safety checks, this approach introduces increased execution time. Moreover, SμV is considered memory-safe and crash-free, but it has not been fully verified yet.

### 2.2. Collective RA

Collective RA protocols aim to provide scalable solutions for attesting large IoT networks. Overall, collective RA schemes differ on whether they consider tree-based or distributed aggregation technique, static or dynamic network topology, one centralized or many distributed verifiers. The collective RA schemes such as SEDA [18], SANA [19], SHeLA [20] rely on the assumption that the network is interconnected and static during attestation. To propagate the attestation requests and aggregate the attestation results in an efficient manner, these schemes construct the network as a balanced binary tree, in which devices have a parent-child relationship. To enable attestation in highly dynamic networks, PADS [21] and SALAD [22] integrate consensus techniques in the remote attestation solutions. Other RA protocols (such as ESDRA [23], DIAT [24]) employ distributed verifiers. Typically, in these approaches, autonomous devices act also as verifiers to attest other devices they interact with. In addition, the RA schemes of distributed IoT services, RADIS [25] and SARA [26], aim to attest, respectively synchronous and asynchronous distributed services of IoT systems. All the aforementioned collective RA schemes rely on hybrid architecture.

### 2.3. Analysis and Surveys on RA

The works presented by Armknecht et al. [27] and Steiner and Lupu [28] are the most closely related to this paper to the best of our knowledge, considering the works in the literature focusing on reviewing software-based RA protocols. The former presents a security framework that formally captures security goals, attacker models and various system and design parameters. The latter focuses on attestation of Wireless Sensor Networks (WSNs), analyzing different RA approaches relevant for WSNs, including software-based attestation schemes. However, these papers do not focus on surveying software-based RA schemes and do not discuss the new IoT opportunities of this research subject. A recent survey presented by Ambrosin et al. [29] provides a comprehensive comparison and analyzes the security properties of the state-of-the-art Collective Remote Attestation protocols that are capable of remotely performing attestation of large networks of IoT devices. Given that this survey focuses only on network RA approaches, software-based RA schemes are only briefly mentioned as they are considered out of scope. Maene et al. [30], and Gross and Sfyrakis [31] survey the hardware-based RA approaches. In particular, Maene et al. [30] present a detailed description and comparison of the security properties and the architectural features of hardware-based attestation and isolation architectures from academia and industry. In addition, the survey presented by Gross and Sfyrakis [31] focuses on reviewing the RA schemes that use a hardware device and cryptographic primitives to assist with the attestation of devices in a network. As such schemes focus entirely on hardware-based RA, the software-based approaches have not been analyzed in these studies. Abera et al. [32] discuss state-of- the-art attestation techniques from the perspective of IoT devices, giving an overview of different types of attestation and discussing their challenges.

To the best of our knowledge, this paper is novel with respect to its focus (software-based RA for IoT) and analysis (research opportunities and open issues). This is shown in Table 1 where we present an overview of the different perspectives on remote attestation of state-of-the-art works and we highlight the main differences with respect to this survey.

## 3. Research Method

In this section, we present our methodology for discovering the existing scientific literature regarding software-based RA approaches. We adopt the research method proposed by Petersen et al. [33], and in the following we elaborate on research questions, search strategy, search process, and study selection.

### 3.1. Research Questions

This work aims to identify and analyze the characteristics of software-based RA protocols proposed in the literature. Thus, we pose the following research questions:

- RQ1: What are the existing software-based RA protocols?
- RQ2: What are the design characteristics and security capabilities of these protocols?

RQ1 aims to provide an overview of relevant software-based RA techniques, while RQ2 points out the features and fundamental differences between the relevant techniques. These questions can then guide us in identifying software-based RA protocols’ opportunities in attesting legacy IoT devices and emerging IoT solutions.

### 3.2. Search Strategy

We use the following PICOC [34] criteria to identify the relevant keywords for our search:

- Population: We consider works in RA domain which propose a software-based approach for resource-constrained devices.
- Intervention: We are interested in works that propose a new protocol or technique on software-based RA.
- Comparison: We compare different approaches based on design features, security capabilities, cryptographic primitives, complexity, required verifier knowledge, etc.
- Outcomes: We present software-based RA techniques, their opportunities and limitations.
- Context: We are interested in any scientific paper in academia that presents a technique or protocol for software-based RA in the context of IoT devices.

Following the criteria mentioned above, we have identified the following keywords for our search: remote attestation, software-based attestation, tamper-proof code, remote software authentication, tamper resistance

### 3.3. Search Process

We performed our search in the DTU Find-it database service, which contains publications from sources like ACM, IEEE, Elsevier/ScienceDirect, Springer, Scopus, Cite-Seer, arXiv and other widely used journals and databases. Since we focus on RA for legacy devices and resource-constrained devices, we used the keyword “tamper resistance” to exclude the wide literature in RA approaches for powerful devices that can support the presence of a specialized hardware, TPM or SGX. Thus, we conducted our search based on the following query: (“software” OR “software-based”) AND (“remote attestation” OR “software-based attestation”) AND NOT “tpm” AND NOT “trusted platform module” AND NOT "SGX". It returned 445 results, and these paper were selected for the following final selection step.

### 3.4. Study Selection

Starting from 445 results, we filtered the papers with multiple phases. Firstly, we identified 67 duplicates, so 378 papers remained for further consideration. Next, we design and apply some exclusion criteria (EC), as proposed by Petersen et al. [33] to filter out the articles.

- EC1: The full-text of paper is not accessible.
- EC2: Paper is not presented in English
- EC3: The proposal of the paper requires additional new or customized hardware.
- EC4: The proposal does not target IoT devices.
- EC5: The paper has been published before 2000.

By applying EC2, EC4 and EC5, the number of considered papers reached 286. After reviewing the title and the abstract, only 41 papers were considered relevant articles in the subset of published materials. In the full-reading phase, we discarded 30 papers, and through reverse snowball sampling, we selected 2 relevant papers. The total number of relevant articles for this survey is 13.

## 4. Background: Software-Based RA on IoT Devices

This section provides the necessary background information on the system model and the adversary model of remote device attestation.

### 4.1. System Model

In general, the system model of a RA scheme consists of the following entities:

- *Prover*. The prover is an untrusted resource-constrained device. The prover performs RA procedure to prove its trustworthiness.
- *Verifier*. The verifier is an external trusted party that validates the integrity of the prover. It is generally assumed that the verifier has access to the prover and knows in advance the expected the legitimate configurations of the prover. It is also assumed that the verifier and the prover interact through a secure communication channel.
- *Network Operator*. The network operator guarantees the secure bootstrap of the software deployed on each prover and the secure key distribution among devices at the beginning of the IoT system operation.

**Figure 1:**
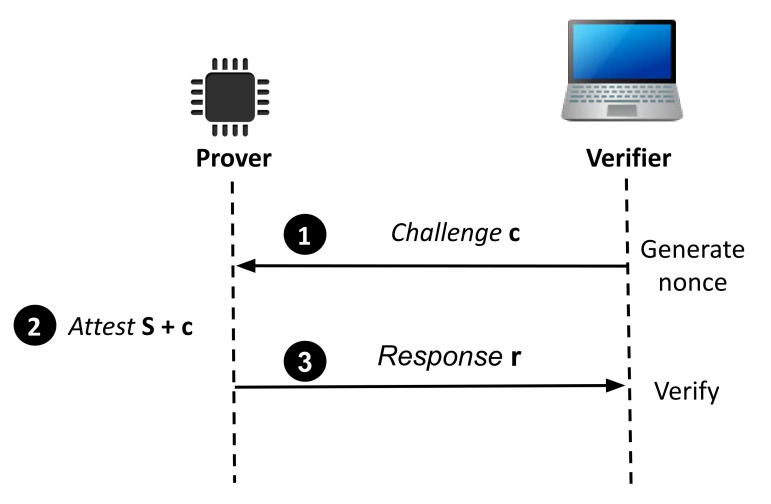
Challenge–response protocol.

Typically, a RA protocol is designed as a simple challenge-response protocol, as shown in Figure 1. The protocol involves two entities: a trusted verifier and an untrusted prover. The attestation starts when the verifier generates a nonce and sends a challenge *c* to the prover (Step ❶ in Figure 1). After having received the challenge, the prover will attest its own device’s state *S*, which is typically a hash or checksum of the device’s memory content. The prover concatenates the attested memory *S* and the received challenge *c* (Step ❷ and sends the computed result in the form of response *r* (Step ❸). Given that the verifier knows in advance the expected the legitimate states (e.g., legitimate hashes or checksums), the verifier is able to compute the expected response locally. The verifier then compares the prover’s response against the expected legitimate value. If the values do not match, the verifier claims that the prover is compromised.

### 4.2. Adversary Model

In the following, we summarize the adversary model of a classic RA protocol. Adversarial actions are also in line with the threat models described by Ambrosin et al. [29], Abera et al. [32], Steiner and Lupu [28].

- *Software Adversary* (${\mathrm{Adv}}_{\mathrm{SW}}$). Injects and executes malicious code on a device. In particular, a ${\mathrm{Adv}}_{\mathrm{SW}}$ will try to generate valid response despite the modification by performing the following adversarial actions.
  1. **Precomputation.** A ${\mathrm{Adv}}_{\mathrm{SW}}$ can pre-compute all attestation operations, independent from the challenge.
  2. **Replay.** A ${\mathrm{Adv}}_{\mathrm{SW}}$ precomputes the attestation result and reports a previous valid response to hide an ongoing attack. A ${\mathrm{Adv}}_{\mathrm{SW}}$ can also eavesdrop a valid response from other devices.
  3. **Memory copy.** A ${\mathrm{Adv}}_{\mathrm{SW}}$ saves a copy of the original memory on the device.
  4. **Data substitution.** A ${\mathrm{Adv}}_{\mathrm{SW}}$ saves only the original data that have been overwritten.
  5. **Compression.** Compresses original data to obtain space for malicious code.
  6. **Impersonation.** A ${\mathrm{Adv}}_{\mathrm{SW}}$ makes a genuine device to send an invalid response and use genuine devices to compute calculations for a compromised device.
  7. **ROP.** A ${\mathrm{Adv}}_{\mathrm{SW}}$ exploits Return Oriented Programming (ROP) technique [35] to execute malicious operations by using legitimate existing code already loaded on the device.
- *Mobile Software Adversary* (${\mathrm{Adv}}_{\mathrm{MSW}}$). A ${\mathrm{Adv}}_{\mathrm{MSW}}$ compromises a device’s software and erases the malware, removing any trace of its presence at the attestation time.
- *Physical Non-Intrusive Adversary* (${\mathrm{Adv}}_{\mathrm{PNI}}$). A ${\mathrm{Adv}}_{\mathrm{PNI}}$ is nearby the device and infers information from the devices, e.g., using side-channel attacks.
- *Stealthy Physical Intrusive Adversary (${\mathrm{Adv}}_{\mathrm{SPI}}$)*. A ${\mathrm{Adv}}_{\mathrm{SPI}}$ captures a device and extracts information from it.
- *Physical Intrusive Adversary* (${\mathrm{Adv}}_{\mathrm{PI}}$). A ${\mathrm{Adv}}_{\mathrm{PI}}$ captures a device and may introduce external hardware on it.

All the software-based RA only attest the program memory, due to the fact that the data memory is unpredictable. Thus, the data memory attacks are considered out of the scope of software-based RA schemes. Due to their software-only design, software-based RA protocols do not consider the detection of ${\mathrm{Adv}}_{\mathrm{MSW}}$, ${\mathrm{Adv}}_{\mathrm{PNI}}$, ${\mathrm{Adv}}_{\mathrm{PI}}$. Additionally, Distributed Denial of Sevices (DDOS) attacks are generally considered out of the scope of any RA protocol, including software-based schemes.

## 5. State-of-the-Art Software-Based RA Schemes

In this section, we present an overview of the software-based RA schemes in chronological order. Their strength and weaknesses are discussed, as well as possible extensions.

### 5.1. Schemes Description

#### 5.1.1. Reflection

Reflection [13] is the earliest attestation found, and it is designed as a simple challenge-response protocol to attest a prover. At the attestation time, the verifier sends two challenges, and each challenge contains a range of memory addresses to attest. Upon receiving a challenge, the prover computes a hash or message digest of the given memory space and its program version. The prover then returns the hash and its version as a response to the challenge. When the verifier has received both responses, it computes the expected hash values and compares them with the the prover’s responses; if they do not match the prover is considered compromised. To guarantee the security, this attestation scheme imposes constraints on the two ranges sent in the challenge. To guarantee that the entire program memory is attested, the ranges should be overlapping and unpredictable. For instance, the verifier randomly chooses two integers $m_{1}$ and $m_{2}$ such that $0\leq m_{2}\leq m_{1}\leq L$, where [0,L] is the range of the programs memory addresses where the program is stored. When the first challenge attests the range $\left[0,m_{1}\right]$, the second challenge attests the range $\left[m_{2},L\right]$ and $m_{2}<m_{1}$, the ranges overlap and many addresses are attested by both challenges.

This protocol is vulnerable to an attacker that first compresses the unused program memory to create space to hide itself, and then during attestation, it decompresses the memory to compute a valid checksum. The paper recommends that the state of the program running on the system be included in the response as a countermeasure to such an attack. The paper also proposes to fill unused memory with random high entropy noise, to be able to attest the memory. The attacker could also hide the original memory values in the devices’s data memory, which is not being attested. To detect this attacker, the paper suggests imposing a time limit within which the prover responds. Additionally, the paper mentions that the protocol is vulnerable to man-in-the-middle attacks because the compromised device could redirect the request to an uncompromised device, intercept the response and use it to pass the attestation. This attack has not been addressed in the paper because it is assumed that the cost outweigh the benefit for the attacker.

While Reflection is the first software-based attestation scheme, it also presents several weaknesses. The paper mentions the memory entropy but it does not consider the entropy of the randomly chosen integers used for the memory ranges. The paper does not explain how the hash is computed and how the memory is traversed. For instance, if the hash is just of the memory’s value and the memory is traversed in a predictable sequential order, it is easier for the attacker to redirect, with an offset, the attestation to where the original memory is stored, computing a valid response. This problem was later tackled in SWATT [4] by not attesting the memory sequentially. In addition, Reflection has a risk of being inefficient. For example, if $m_{1}=L$ and $m_{2}=0$, then the entire memory is attested twice, which is unnecessary, and if $m_{1}$ and $m_{2}$ is just close to the boundaries of the range, it means most of the memory is attested twice. From an IoT point of view, attesting memory twice during the same run, uses precious battery, bandwidth and prevents devices from doing their regular operations. Depending on how often devices are attested, the attestation could result to be a costly operation.

#### 5.1.2. SWATT

SWATT [4] is a simple challenge-response protocol, similar to the one shown in Figure 1. SWATT computes the response by performing a checksum of the memory. It uses a pseudo random number generator (PRNG) to iterate over the memory in an unpredictable order. In this way, the attacker has to check every memory access during the attestation, in order to redirect the memory access to where the original code for that memory location is stored. The prover receives the seed for the PRNG in the challenge from the verifier. To ensure with high probability that the device is not compromised, SWATT traverses O(nln(n)) memory addresses, where n is the number of memory addresses, in which the program running on the device is stored. This value comes from the Coupon Collectors Problem, which states that with this number of memory accesses, it is likely that every memory address in the device is accessed at least once. With every memory address accessed at least once, the attacker cannot store unexpected values in the attested memory.

To prevent an attacker from computing a valid response on the fly during the attestation execution, by redirecting the attestation to memory regions where the attacker has stored the original values, SWATT requires a strict running time of the attestation procedure. To meet the upper bound time limit of attestation procedure execution, the SWATT attestation code must be fully optimized. Due to PRNG properties, the attacker needs to insert *if* statements into the attestation code to redirect the check to where the original code is stored. Thus, any injected malicious code will result in a measurable delay. Another requirement for the timing check, is that the attestation response cannot be computed concurrently, i.e., different parts cannot be computed at the same time. If this was possible, a number of devices would be able to collude to compute the legitimate response within the permitted time. To prevent the concurrent computation, each checksum and memory address is dependent on the previous one.

The fact that SWATT relies on a strict time limit and optimized function execution is considered a drawback. In particular, as devices become powerful and algorithms get improved, the SWATT algorithm would need to be continuously updated, to ensure that no faster implementation exists. Furthermore, it presents a problem when used in networks with unpredictable delays. In this case, the allowed time might be too long, which would allow attackers to remain undetected. In contrast, if the allowed time is not long enough, devices might be considered compromised even if they are not.

#### 5.1.3. Pioneer

Pioneer [5] is a RA scheme similar to SWATT [4], but it focuses on legacy devices with more processing power and more memory. Pioneer differs from SWATT in that it includes more information in the checksum. Unlike SWATT that includes only the values of the memory addresses in the checksum, Pioneer also includes the program counter and the data pointer in the checksum, in order to detect memory copy attacks. Furthermore, the jump locations for jump instructions are also included in the checksum, to detect illegal jumps.

Pioneer is a two-step challenge-response protocol. First, it computes a checksum of the checksum code to verify that it works correctly. Then, it computes a hash of the executable that is being attested. The verifier checks both the checksum and the hash to confirm the executable is trusted. Pioneer also differs from the other schemes presented, in what is being attested. For devices with large amounts of memory and many different programs, Pioneer only attests one executable, which is then used to provide a route of trust for that executable. Pioneer requires the same strict time constraints as SWATT and uses the same PRNG to select memory addresses.

#### 5.1.4. PIV

The Program Integrity Verification (PIV) [36] is a RA scheme based on a randomized hash function (RHF). The RHF used is a multivariate quadratic (MQ) polynomial. The MQ polynomials have successfully been used as a one-way trap-door function. The special MQ polynomial characteristic PIV uses, is that the same hash value can be computed from both the memory content and a special digest of the memory content. That is, running the RHF on both the program and a special digest of the program, will produce the same value. The MQ polynomials have the following special characteristic. If a program x is split into n blocks $\left[x_{0},\ldots ,x_{n}\right]$, where each block is a m×1 vector $x_{i}={\left[x_{i,0},\ldots ,x_{i,m}\right]}^{T}$, then the special digest is the m×m matrix $X_{l}=x_{l}x_{l}^{T}$.

The digest is clearly related to the program x. During the verification the verifier computes the hash *Y* from the digest $X_{l}$ by

$$Y=H\left[\sum_{l=1}^{n}g_{l}X_{l}\right]H^{T}$$

and the prover computes the hash *Y* from the program *x* by

$$Y=\sum_{l=1}^{n}g_{l}\left(Hx_{l}\right){\left(Hx_{l}\right)}^{T}$$

where *H* is the random hash function, $g_{l}$ is a cryptographic key and *n* is the number of blocks the program consists of.

PIV is a typical challenge-response protocol, but it sends more messages to authenticate the verifier. The protocol starts either when a device tries to join the network, or when an intrusion detection mechanism flags the device as potentially compromised. When a device tries to join the network, it first finds at least a verifier and an authentication server (AS), and then, it asks the AS to authenticate the verifier. Once it has found an authenticated verifier, it will ask that verifier to attest its code. The verifier sends the randomized hash function *H* and the key $g_{l}$ to the device. The device computes the hash from the program and sends the hash as a response to the challenge. If it does not match the verifier’s expected checksum, the device is considered compromised and cannot join the network. Suppose the device is already on the network and the intrusion detection system flags it as suspicious. In that case, the verifier can initiate the protocol by sending the hash function and the key. The device still authenticates the verifier with the authentication server.

Unlike Reflection [13] or SWATT [4], in PIV [36], the verifier does not store the programs but only some digest of program blocks. Since programs from different sensors might use the same blocks, this means the verifier might need to store less information. PIV claims to be efficient as it does not attest devices often: a device is attested when it joins and when an intrusion detection system (IDS) flags a device as possibly compromised. Since how often PIV will attest devices depend on the IDS, it might often run if the IDS is very sensitive or if many attacks are triggering the IDS to attest the devices.

#### 5.1.5. Self-Modifying Code

Shaneck et al. [37] propose a RA protocol that improves the strict time constraint of SWATT [4]. The key idea is to make it difficult for attackers to insert conditional offsets into the read statements of the attestation code by making the code different for every attestation. In order to achieve this, the attestation code is required to be fresh and unpredictable. The freshness is to avoid replay attacks, while unpredictability prevents pre-computation. In the proposed solution, the verifier sends the attestation code over the network. For this, the code should be small not to introduce high communications overhead during the attestation procedure.

The scheme focuses on making static program analysis of the attestation code difficult and time-consuming, preventing attackers from computing a valid response within the expected time limit. Unlike SWATT that relies on a strict time constraint and optimized attestation code, this scheme has a looser time constraint and does not rely on the optimized code. In particular, the scheme relies on code obfuscation to prevent any attacker from analysing the code and computing a correct response in time. The solution suggested is intended to add more delay from an attack, to ease the strict time limit, allowing for more delay in the network. The proposed time constraint for this attestation scheme is (2×r)+e+Δ, where *r* is the transmission time of the challenge and response, *e* is the expected execution time of the attestation code, and Δ is the variable time. Δ is based on the delay to an attacker, and hence is the allowed network delay.

This scheme is a challenge-response protocol, with the addition of using encryption and message authentication codes (MAC). The challenge contains the actual attestation code, which has been encrypted and sent along with a MAC of it. After the prover has verified the MAC, it decrypts the code and loads it into the program memory to execute it. When the verifier constructs the attestation code, some form of randomness is used to provide freshness and make the code unpredictable.

This scheme is an improvement over SWATT when it comes to maintainability. Since SWATTs attestation code has to be fully optimized, it needs to be continuously checked and updated so that no attacker could have a faster version with conditional offsets. This is not required by Self-Modifying Code, as the code is a fresh version provided by the verifier at each attestation. This means that the verifier has to send more data, which could be an issue for sensor networks, where the bandwidth is low. For example, if the sensor is somewhere remote and running on a battery, then this communications overhead might be too costly.

#### 5.1.6. Proactive

Proactive Code Verification [38] is an attestation scheme trying to improve upon SWATT [4]. Like the Self-Modifying Code [37] scheme, it aims to solve the strict time constraint imposed by SWATT. Rather than changing the attestation code, Proactive focuses on filling the memory with random values to prevent the attacker from hiding the original values or the malicious code. This protocol is a classic challenge-response protocol attesting the memory by computing a checksum over the content. It suggests adding an identifier for the verifier to the request, allowing the prover to authenticate it. In the request, the prover receives a seed, same as SWATT. Instead of using the seed to attest memory in random order from the randomly generated values, Proactive uses the seed to generate random values used to fill the empty memory. Additionally, instead of responding with only the program memory checksum, Proactive includes the data memory in the checksum. To verify the checksum, the verifier needs the data memory content, which the prover sends in the response along with the checksum.

This scheme also has a time constraint, but it is not required to be as strict as SWATT. As the entire memory is attested, an attacker cannot use unused memory to calculate a valid response from original values. Moreover, in Proactive, the values used to fill the memory rely on the previous values. The attacker will therefore not generate the values easily and hash them. Since Proactive fills the memory twice, the attacker cannot simply calculate the chains, as the results propagate through the values for all the filled memory. Proactive includes the data memory in the checksum and sends the memory content in the response to introduce more data to be sent over the network. Since the verifier does not know the legitimate state of the data memory, it accepts the response content despite potentially malicious code that it may include.

Proactive’s improvement on the time constraint of SWATT is due to the fact that it would take an attacker a long time to compute each block of memory to be attested, without overwriting its own malicious code. The attacker would have to compute each block of memory from the beginning since each block relies upon the previous blocks. Because the memory is filled twice, it means the first block relies on all the other blocks.

#### 5.1.7. Distributed

Yang et al. [39] propose two different distributed attestation schemes. The idea behind both of them is to remove the need for a trusted verifier as in other attestation schemes. In addition, an efficiency enhancement for SWATTs [4] pseudo-random memory traversal is suggested at the cost of security. This scheme relies on SWATT but with the difference that it works on memory blocks instead of cells. Instead of computing the checksum by iterating over each memory address and updating the checksum, the proposed block-based approach handles a block of cells at a time, which are Xor-ed together to compute the checksum. Such an approach results in fewer iterations since each iteration handles more memory. If the block size is set to one, then it is the same as SWATT. If the block size is the memory’s size, then the checksum is computed in one iteration, where all memory is Xor-ed together. A block size equal to the size of the attested memory is a security risk, as it makes it easy for an attacker to store the computed checksum since there is no actual traversal. Therefore one has to be careful when choosing the block size.

In the first scheme, one device is elected and acts as the verifier. Majority rule verifies the attestation response in the second scheme. In both schemes, the memory is filled before the devices are deployed. The memory is filled with pseudo-randomly generated noise. It uses RC5 in CTR mode to fill the memory, which is a common hash algorithm running in counter mode. Counter mode means that the values generated are not reliant on the previously computed values. Instead, the computed values depend on a counter, which is encrypted. That means, if the seed and the counter value are known for a specific block, then it is possible to compute the value without having to compute any other values. This is less secure, as the values are not mixed together the same way as in CBC mode. These schemes do not have a powerful verifier with lots of memory to handle the CBC mode, which is why the CTR mode was chosen, as it uses less memory.

In the first scheme, the idea is for the provers neighbours to have the seed for the memory filling as a shared secret. This means the neighbours can recreate the values used to fill the memory to compute a checksum to attest against. Once the device is deployed, it finds its neighbours and then sends a part of the seed together with a hash of the seed to each neighbour. The secret sharing relies on a (n,k) threshold, where *n* is the number of neighbours and *k* is a value representing a trade-off between security and performance. This has to do with defining how many neighbours the secret has to be shared between to be secure. The threshold *k* is defining how many shares are needed to restore the secret. The higher *k* is, the more shares are needed, making it more difficult to obtain the secret and requiring more communication when a device actually needs to get the secret.

The second scheme does not rely on a cluster head but a democratic process. Instead of sharing the seed amongst the neighbours, each device is loaded with *n* challenge-response pairs before it is deployed, where *n* is the number of expected neighbours. The number of memory traversal iterations is configurable and is a trade-off between security and performance. When the device is deployed, it finds its neighbours and sends a challenge-response pair to each of them. Upon a device is chosen for attestation, each neighbour sends its challenge sequentially. The prover computes the responses with the PRNG block-based memory traversal. The neighbours then vote on the result and the majority rules.

In the first scheme, the protection of the seed is critical. If the seed is compromised, then the attacker can pass any attestation for the device to which the seed belongs. That means the attacker needs to obtain *k* or more secret shares, e.g., by compromising the neighbours. Another possibility the attacker has is to be cluster head during attestations, then compromise those devices it attested. This way, it will have the secret seed for them, letting them pass attestation and do the same when they are cluster head.

The second scheme does not have the weakness mentioned above. Instead, it is a democratic scheme, so if the attacker has compromised enough devices, the attack will not be detected. This means this scheme relies on the probability of each device being compromised and detection mechanism to detect the attack before too many devices are compromised. Furthermore, the second scheme could end up being extremely inefficient if focused too much on security. If a prover has many neighbours and it has to traverse the entire memory for each neighbour, it would prove extremely inefficient. The first scheme has the advantage here since only one attestation execution is performed.

Furthermore, the schemes presented here have a high communications overhead, which can be costly on devices’ power consumption, especially when considering IoT battery-powered and resource-constrained devices. The second scheme introduces communications overhead and computational overhead, as the prover might have to attest its whole memory several times.

#### 5.1.8. Memory Filling

AbuHmed et al. [40] present another attempt at using memory filling to overcome the time constraints imposed by SWATT. Filling the memory is proposed to be done in two different ways, together with two attestation protocols. Furthermore, an alteration is suggested to the block-based pseudo-random memory traversal used in the Distributed [39] schemes. The pre-deployment memory filling scheme of Memory Filling [40] is very similar to that of [39]. Both schemes use RC5 in CTR mode to fill the memory of the devices before they are deployed. The difference is that in [40] a trusted verifier does the attestation. The change suggested to the PRNG is to make the block size dynamic in the algorithm. This involves calculating a new block size in each iteration. The paper suggests having the block size be a function of the output from the RC5 hash function but does not suggest any requirements.

The paper suggests two attestation protocols. The first one is the same basic protocol as any other attestation scheme, but with ids and encryption for authentication and protection of the messages during communication. It also sends a nonce from the verifier to the prover and another nonce from the prover to the verifier. This is a basic and common way to provide freshness proof for both sides. The second attestation scheme adds a timestamp to the attestation request, in order to prevent replay attacks.

The post-deploy memory filling could be useful if some data is collected and stored in the program memory, to be used for the devices normal operation. It would notify the verifier about the seed and memory. The block-based pseudo memory traversal algorithm is an interesting suggestion to make it less predictable, but it is not justified from a performance or security perspective. It would randomly increase performance or security, depending on whether the block gets bigger or smaller. In the end, it will depend on the number of iterations done. The dynamic block size $O(\frac{n\;ln(n)}{b})$ is not a good measure for number of iterations, as *b* is not fixed. The likely scenario is to run O(nln(n)) iterations to be on the safe side.

The memory filling of these schemes is using RC5 in CTR mode. The reason Distributed [39] used CTR mode was because of the better performance on memory usage. This choice was made because the code was running on other resource-constrained devices, where memory usage performance matter. In Memory Filling [40], a verifier, which is likely a more powerful machine, does the attestation. The prover does not need to fill the memory again, it just needs to compute the checksum, so it is unaffected by CTR vs. CBC mode. The verifier is likely to have the memory to generate the expected memory with RC5 in CBC mode rather than in CTR mode, thus being more secure at the cost of some temporary memory usage. After the attestation, the memory with the expected memory content can be overwritten and used for the next attestation.

#### 5.1.9. USAS

USAS [41] is a RA scheme that aims to improve the time and power performance of Distributed [39] and SWATT [4]. Thus, it relies on two layers of attestation, where only one layer is dependent on a PRNG, improving the performance of the second layer. Even though the scheme is mostly based on Distributed [39], it uses a trusted verifier, rather than the distributed model of [39]. The focus is on the time and power performance of the attestation.

In the scheme presented, I- and F-devices distinguish the two layers. The I-device is the initiator, which means that it is where the attestation starts. The F-devices are the followers, which are in the second layer of attestation. The devices to attest, both the I-device and the F-devices, are picked randomly by the verifier to ensure unpredictability. The basic idea is that the verifier sends a random challenge to the I-device. The device computes a checksum from the challenge. The I-device then sends the checksum to the F-devices, instead of to the verifier. The F-devices use the checksum to compute their checksum. The F-devices send their checksum to the verifier, and the verifier compares them with a locally computed expected checksum.

The memory of the devices is filled before they are deployed, similar to previously described schemes. The challenge message contains the seed for the PRNG and the seed used for the random noise generation used to fill the memory. The devices have a hash of the seed stored, which they use to authenticate the challenge. The I-device receiving the first challenge uses RC4 to generate random memory addresses for the attestation. This is the attestation algorithm used in SWATT [4]. The resulting checksum is sent to all the selected F-devices. This is where the the most significant difference comes. Instead of using RC4 to generate a random address for attestation, the F-devices use the I-device checksum for generating addresses. The noticeable part of the algorithm is that the memory address to be attested is computed from the I-device checksum combined with its checksum, which it is currently computing. It still iterates over the memory O(nln(n)) times, but it does not run RC4 on every iteration to generate a new random address. The RC4 value is, therefore, also not used to update the checksum. When the verifier compares the checksums from the F-devices with its own locally computed checksums, it also verifies the checksum of the I-device each time. This is because the checksum from the I-device is used to compute all the other checksums. If the checksum of the I-device would not pass, then none of the other checksums would pass. That means if at least one F-device passes the attestation, then the I-device also passes. However, it does also mean that if the I-device is compromised and cannot pass, then all the F-devices will also fail even if they are not compromised. This means one attestation round is not enough to say that a F-device has failed if all the F-devices failed. If just one F-device passes, then the I-device can be trusted, and any F-device that fails can be considered compromised. If all checksums’ validation fails, there will be another round necessary to check if it was because of the I-device. Thus, the devices will be re-attested until at least one device passes.

Even if the performance might be better for each attestation, it depends on how likely it is that the I-device is compromised. Each time the I-device is the compromised device, all the F-device attestations are useless, and SWATT would have been more efficient. There is also always the possibility that the I-device is not compromised, but all the F-devices are, depending on how many F-devices there are in each attestation.

A security risk with this scheme is that the challenge includes the seed used to fill the memory. This means an attacker can obtain the seed and generate the expected memory content on the fly. Since there is no time constraint imposed, the attacker has plenty of time to generate a valid response. This seed is only used to authenticate the verifier and thereby the challenge. Instead of sending the seed, the hash value should be sent not to disclose the actual seed. However, if an attacker eavesdrops and learns the message, be it the seed or a hash of it, then the attacker can authenticate as the verifier. This means that it is crucial having a secure authentication protocol with messages encrypted with a strong enough encryption.

#### 5.1.10. DataGuard

DataGuard [42] focuses on preventing overflow attacks in the data memory. Unlike the other schemes, it provides security assurances for the data memory’s integrity, without overwriting the memory content. As long as the dataguards cannot be reconstructed, the scheme will detect any overflow attack, which has happened since the last attestation.

The way the scheme prevents these kinds of attacks is by introducing new variables called dataguards. The dataguards are appended to the end of the variables used by the program. The point is, that memory is always filled in one direction, so if more memory is filled than is allocated to the variable, it will fill the dataguard. Since the dataguard has been changed, the device will not be able to pass attestation. To prevent an attacker from passing attestation, the dataguards are not allowed to be able to be recreated, without some secret information, which is not stored in the device.

The dataguards are initialized from a secret *e* and a nonce provided by the trusted verifier. The first dataguard is a hash of the two values from the verifier and the initial value of a counter *c*, that is the initial dataguard is $H\left(e,nonce,1\right)=dg_{0}$, where $dg_{0}$ is the dataguard and 1=c is the initial value of the counter *c*. Both *e* and nonce are fresh and randomly chosen, thus providing entropy and making if difficult to guess them. The security of the dataguards, relies on the attacker not knowing *e* and nonce, since knowing them would allow the attacker to compute all valid dataguards for passing attestation. When a new dataguard is computed the previous dataguard $dg_{i-i}$ is updated as well. The previous dataguard becomes $dg_{i-1}=H\left(dg_{i-1},c+1\right)$ and the new dataguard is set to $dg_{i}=H\left(dg_{i-1},-\left(c+1\right)\right)$. Note that the new dataguard is computed from the old value of the previous dataguard, not from its new value. The dataguards computed are never deleted or removed. If they are not used anymore, because they belonged to a temporary variable, then they are stored in a list of dataguards as they are still needed during attestation.

The attestation protocol of this scheme is again a challenge-response scheme. The verifier sends a challenge to the prover. The prover computes a checksum of the dataguards which it sends, together with the number of dataguards m computed, as a response to the challenge. The verifier then computes the expected value locally, as it knows both *e* and nonce. If any of the dataguards do not have the expected value, then the checksums will not match, the attestation fails, and the device is considered compromised.

It can be prevented by attesting that the program memory has not been altered or by using hardware such as a TPM for the dataguard generating program. Furthermore, it is interesting that the verifier sends both *e* and nonce during the initialization, but it has no freshness in the actual attestation challenge. If *e* is a freshly generated secret, it provides the freshness, making nonce redundant. On the other hand, it increases the entropy, making it more difficult to guess the initial dataguard.

Since there is no freshness in the attestation challenge, an old computed response can be used. That is the attacker can compute a checksum of the current dataguards and save the counter value. Then, the attacker can overwrite all the current and new dataguards. When attestation time comes, the attacker sends the computed checksum with the counter as a response and passes attestation.

If this is done as part of the update operation, the attacker will also gain the new secret and nonce, thus being able to compute valid dataguards.

This attestation scheme relies a lot on the dataguard generating program not being compromised but does not attempt ensuring it, either by attesting it or using hardware. It might also run into some performance issues. According to [42], 150 dataguards can be stored in the list if it has 1 K memory. Furthermore, it says it can compute a dataguard in 0.01 s. It does not mention the communications overhead from update operations. The communications overhead depends on the program, as it depends on how often a dataguard is generated. If the program uses many local variables, it will generate many dataguards. Let us say the program generates 150 dataguards every hour and stores 150 dataguards in the list, then it will run the update operation with the attestation run every hour. This could be costly for battery-powered and resource-constrained IoT devices.

#### 5.1.11. Lightweight

The attestation scheme presented in Lightweight [43] is very similar to the second scheme of Distributed [39]. They are both distributed schemes, in that they do not use a trusted verifier. They also both distribute the responses for an attestation challenge. Lightweight [43] uses an initialize phase, where the responses are distributed amongst devices. It works by each device filling some attestation memory with checksums of values from other devices registers. For all registers of a device to be checked and to avoid one register to be checked by multiple devices, each register is only distributed to one other device. For this, this work assumes the memory is divided into program memory and attestation memory, both still being static memory. The two memories should be equal in size, and if the program part of the memory is not filled by whatever program is on the device, it should be filled with random noise. The initialization phase is complete when all devices have filled their attestation memory with checksums of register values from other devices.

The attestation protocol involves only two devices. Let us say device *a* initiates the attestation. It then picks one register from program memory and one from the attestation memory, both to be attested. It then collects the equivalent values from the devices that store them. The register from the program memory stores an actual value, so the device collects the corresponding checksum form the device that stores it, computes the checksum for its register and compares the two. The register in the attestation memory contains a checksum, so it collects the original value from the device it got the value to compute the checksum from, computes the checksum of the value it just collected and compares the two. If either of these two checks fails, both devices are terminated.

In Distributed [39], the responses to challenges are also distributed amongst devices. Each response does, however, consider more than one register. Furthermore, it does not terminate two devices on one failed attestation, as only one device is being attested by many, done through a democratic process. Lightweight assumes the program and attestation code are stored in read-only memory to prevent it from being modified. The reason it terminates both devices, is because it is attesting both devices together on the same checksum one to one and carried out by one device. This also makes it susceptible to DoS attacks, which is considered out of scope in [43]. One issue here is the assumption that a bad device will terminate when failing. This is assumed to happen because the code is stored in a read-only memory and cannot be changed. Furthermore, this termination procedure means that an uncompromised device is terminated for each device which fails attestation. This could mean that many uncompromised devices are terminated, which is undesirable since it could interrupt operations relying on them.

Another consideration of this is how the whole scheme is affected when many devices are terminated. If a register checksum is stored on another device, then that register cannot be attested if the device is terminated. If enough devices have been terminated, the attacker may have enough unattested memory to avoid detection. However, it requires the attacker to know which registers were in the terminated devices, which could prove difficult. Furthermore, Ref. [43] does not explain how it deals with a device picking a register, which is stored in a terminated device, to attest.

Another interesting question for this protocol is how it would work in a more heterogeneous network. The current design focuses ona homogeneous network, where every device has the same memory size, in order for all registers to be stored on another device. Nevertheless, in a heterogeneous IoT network, this might not be possible. As an example lets consider a network with 2 devices *a* and *b*. Device *a* has a memory size *n* and device *b* a memory size *m*, where n<m. If the memories *n* and *m* are both equally split in program memory and attestation memory, then device *b* could store all checksum values of device *a* registers, but device *a* would only be able to store $\frac{n}{2}$ of device *b* registers. Thus, not all registers of device *b* would be able to be attested. Even though this small example only has two devices, the problem gets more prominent in larger networks with even more different devices. It also gets more complicated if the device’s memory is not split evenly or not split equally across the same type/model of the device.

#### 5.1.12. LRMA

Low-cost Remote Memory Attestation [44] is building on top of SWATT [4]. The scheme addresses the strict timing constraints imposed by SWATT. It modifies the time handling to allow for attestation of devices, not in direct contact with the verifier. Furthermore, it adds a probabilistic risk level, which is used to determine the sequence and frequency of attestation. These two parts are disjoint, so the time handling will be described first and then the risk level.

In LRMA devices any number of hops away can be attested. In most attestation schemes, only devices in direct communication with the verifier are considered. Since [44] uses SWATT as the base attestation scheme, they need to consider the time constraints in a setting with several network messages which can be delayed. LRMA handles this by having the verifier receive the network delay for each hop in the response. It then uses these delays to compute an average and use the average to estimate to estimate the actual attestation time.

LRMA [44] introduces the use of risk levels to determine the frequency with which a device is attested. This means that a device with a high-risk level is attested more often than a device with a lower risk level. The risk level is calculated as the sum of failed attestations in some $n_{r}$ recent attestation runs. When determining the frequency $T_{i}$ at which a device is to be attested, it uses the calculated risk $R_{i}$, the average risk level $\tilde{R}$, a time unit *T*, a scaling factor β and a constant φ.

$$T_{i}=\frac{R_{i}+\varphi }{\tilde{R}+\varphi }\times T\times \beta$$

The verifier uses this frequency to determine the devices next attestation time. This is done by picking a random value between zero and $T_{i}$ and adding it with the sum of the previous frequencies. If zero was picked randomly and the sum of previous frequencies was not added, it would mean the device should be attested right away; thus, the sum provides an offset. Interestingly it is a sum and not an average, which means the offset increases as time passes.

On the other hand, if a device is still terminated if it fails attestation, then the risk would reflect how often a device is compromised. Thus, saying something about that particular device having some vulnerability that needs to be fixed if it has a high-risk level. However, it might take a while for a compromised to be fixed and be allowed back on the network. When the device gets back on the network, it needs to be identified as the same device. Since it is unknown when a device will be back on the network, the next attestation time should not be determined until it is connected again.

Another way the risk level could be used is to only use it for the timeout check. If the attestation fails because the checksum does not match, then the device is terminated. However, if the time used check fails, then the risk level and attestation frequency are updated. There should then be a threshold for the risk level, so if a devices risk level surpasses the threshold, it is terminated. This will let the average time measurement of the network delay have a little more time to compute a more accurate average. However, it will also give an attacker more time to remain undetected on a device, thus increasing false negative attestations.

If the compromised device is not terminated, but just attested more often, it also introduces much bias in the performance calculations and experiments. In [44], results of some experiments on its performance are shown. One of the experiments shows that LRMA has a lot more successful attestations and no undetected attacks after some time. In these experiments, the successful attestations are attestations that correctly detects an attack. However, if the compromised device is never terminated, and instead the compromised devices are attested more often, then at some point only the compromised devices are attested and fails because of the same attack.

Since the total number of attestation is same, it would also mean that after some time, some devices might never be attested, thus being a perfect target for an attacker in case they would learn this. By collecting and considering the network delay, this scheme should reduce the amount of false negative attestations. These false negatives would be attestation that fails because they used too much time, but the time used was actually because of network delay, not because the device was compromised.

### 5.2. Summary of RA Schemes

Table 2 and Table 3 present the main characteristics of the RA schemes described in the section above. While the protocol complexity is based on either the amount of *n* memory addresses to be attested, some schemes, however, also depend on a block size *b* or the number of neighbours *m*. Unlike others, Dataguard [42] depends on the number of variables *v* in the program.

The method in Table 2 refers to the type of attestation technique that has been adopted. The software-based RA schemes either impose a strict time constraint like SWATT [4], Pioneer [5], LRMA [44] or fill the memory with random incompressible noise like the works in [39,40,43]. The strict-time approach aims at preventing the attacker from redirecting the attestation codes read operations to the empty memory, where the attacker has stored the original program. Due to network delays, it might be challenging to predict or assess the time of attestation routine. Thus, these RA approaches assume that the unused memory is left empty with some special values, e.g., 0. The memory-filling approach fills the device’s memory with random noise. Thus, it prevents the attacker from using the memory because the attacker will not be able to recreate the original noise value used to fill it. The method also relates to the time column, which gives a quick overview of how accurate the time measurement for the attestation needs to be.

It is also interesting to note the different kind of hash functions the schemes suggest to use (as shown in Table 3). Some newer schemes still propose RC4 even though a never version RC5 has come out. In contrast, other schemes propose heavier hash functions, like SHA-1, to use because they do not run the hash algorithm on a low powered IoT device on every attestation.

## 6. Opportunities of Software-Based RA Schemes

Software-based RA protocols have been abandoned in the most recent RA proposals as they are considered deprived of necessary security guarantees. However, the lightweight design of such protocols could be of great value for various already-deployed IoT solutions or new commercial IoT products. In the following, we discuss some opportunities that software-based RA approaches bring in enabling attestation on different categories of very lightweight IoT devices.

### 6.1. Legacy Devices

With the large number of IoT devices deployed over the past years, many IoT devices currently in use are legacy devices. Most legacy IoT devices were designed to operate unconnected, standalone, and the adoption of novel security solutions are often impractical for such devices. Considering the unique characteristics of legacy IoT devices that typically lack complete and accurate documentation, it becomes crucial to bring RA’s benefits to such legacy devices without disrupting their existing operations. In this context, the adoption of hardware or hybrid RA schemes requiring specialized hardware support or customized hardware configuration is impractical for legacy IoT devices. In contrast, the software-based RA approaches are suitable for legacy devices as they rely only on software. Even though software-based RA protocols are vulnerable to sophisticated attacks as discussed in Section 7, software-based RA protocol could still provide some degree of integrity guarantees in these devices. Under certain assumptions such as legacy devices deployed in a private and relatively-small network, the software-based approaches such as SWATT [4], Pioneer [5] and LRMA [44] are a promising solution for the missing security mechanisms present on resource-constrained legacy IoT devices.

### 6.2. Battery-Free Devices

Europe has recently entered into the green transition, which aims at lowering global energy footprint towards achieving the ultimate goal of being climate-neutral by 2050. As a result, the deployment of battery-free IoT devices [12] is expected to be increased in the upcoming years. In this context, the RA protocols that rely on customized hardware not cause an increased cost and size of any resource constraint IoT devices and deviate from the initial core objective of the original energy-harvesting design of battery-free IoT devices. While typically the IoT networks of such tiny devices adopt correlated information to detect compromised devices, such battery-free devices could benefit from software-based RA schemes as an integrity check mechanism. However, the software-based RA protocols that perform expensive computational operations and rely on strict time constraints could be heavy for such devices. The most suitable protocols for energy harvesting devices could be the software-based RA protocols that rely on loosely time constraints listed in Table 2 such as [40].

### 6.3. Fog Computing

Due to strict time constraints, software-based RA schemes have been considered limited to a one-hop network setting and unsuitable for attestation of large networks with multi-hop distance between the verifier and provers. However, with the emerging paradigm of Fog computing (https://www.openfogconsortium.org/ (accessed on 31 December 2020)), there comes the opportunity to introduce single-hop attestation schemes between these devices and a connected Fog node, that can act as a verifier. The software-based RA schemes have been considered impractical due to the strong assumptions of the required verifier’s knowledge to validate the legitimate state of IoT devices, for instance knowing the exact hardware configuration. Table 4 presents an overview of the required knowledge by the verifier. In a Fog computing infrastructure, each Fog node serves as a distributed verifier, the assumption that each Fog node has all the required knowledge of the devices connected to the Fog node seems realistic. Thus, each Fog node may attest its device by performing a software-based RA scheme. However, software-based RA schemes are challenging in mobile networks in which devices frequently join and leave different Fog nodes.

### 6.4. IoT Applications

Software-based RA schemes serve as building blocks for other crucial software-based security mechanisms such as key establishment [45], security software update [46], recovery [47] and secure erasure [48]. With the IoT devices playing a remarkable role in many domains such as healthcare, vehicles and transportation systems, industrial appliances, and smart homes, the cutting edge of security is continually being pushed. Recent works in the literature have integrated RA with Blockchain to provide stronger security guarantees (e.g., decentralization, traceability, anonymity and non-repudiation) for critical real-time infrastructures such as Vehicle-to-Vehicle communications [49]. Other promising applications include the trustworthy collaboration among Automated Guided Vehicles in the mobile and collaborative Smart Factory context [50].

## 7. Open Issues

### 7.1. Key Establishment

Secure and efficient key establishment mechanisms are crucial for any RA approach in the IoT domain, and they are especially critical for software-based approaches. Generally, the software-based RA schemes rely on the assumption of a secure communication channel between the verifier and prover. However, this is an unrealistic assumption considering that the software-based RA approaches do not provide the required hardware protection for key storage to guarantee that the received messages originate indeed from the intended prover. Thus, a secure communication channel between prover and verifier is not guaranteed, and key establishment in software-based RA schemes remains an open research challenge.

### 7.2. Undetected Attacks

Software-based RA schemes provide different security guarantees. Table 5 presents an overview of the main attacks that the existing software-based RA schemes can detect. All of the attestation schemes are open to attacks where the attacker hides malicious code or original values in the data memory. In addition, software-based RA protocols are vulnerable to attacks based on Return Oriented Programming (ROP) and data compression, as is demonstrated in the work [51]. In the ROP attack, the attacker inserts a command in the function receiving the attestation request from the verifier. The inserted command redirects the control of a procedure which cleans up the memory. The malicious code moves all its code from the program memory to the data and external memory. It then deletes itself from the program memory, such that there is no malicious code left in the program memory.

The other well-known attack against software-based attestation is the compression attack. In this attack the original program is compressed, opening up space for the malicious code. During attestation the original code is decompressed on the fly, allowing the attacker to produce a valid attestation response and not getting discovered. Many schemes use memory filling technique and assume that the memory is filled with incompressible random noise. One interesting avenue to explore to defeat this attack is for the attestation routine to compress the program, fill the memory and then attest the memory, after which the program can be uncompressed. Thus, preventing the attacker from having empty memory to evade detection during attestation.

The vast majority of attestation mechanisms do not pay much attention to the verifier’s security. They are developed under the assumption that the verifier can always be trusted. However, an adversary can take advantage of this assumption and impersonate the verifier to perform Denial of Service attacks targeting honest devices.

### 7.3. Interruptability

Traditionally, all RA protocols, including software-based RA approaches, get executed randomly at unpredictable times, and the Prover has to stop the regular operation to perform attestation. Thus, RA requires uninterrupted power supply during attestation, and it prevents Provers from performing their regular operations during RA execution. These RA characteristics might be intolerable for IoT devices that perform time-critical operations or devices that work under intermittent connectivity. Thus, interruptability remains an open research challenge in RA domain.

### 7.4. RA in Medical IoT Devices

Healthcare applications remain a challenging domain for running RA protocols. Medical Internet of Things (mIoT) consists of interconnected heterogeneous medical devices enhanced with sensing and actuation capabilities. In particular, mIoT includes: (1) implantable medical devices which are medical devices surgically placed inside the human body to monitor or treat a medical condition, and (2) Body Area Networks which are wireless networks of wearable computing devices that provide remote healthcare monitoring of the patients. In general, mIoT devices are resource-constrained, with heterogeneous hardware and software platforms, and limited processing and storage resources. Furthermore, existing medical devices pose some specific security issues such as lack of security engineering, customized proprietary interfaces and supply chain vulnerabilities. Thus, considering these limitations, it becomes very challenging to deploy RA protocols in such medical devices. In this context, software-based RA schemes are promising because they can be constructed to run on many low-end devices without extra hardware. However, due to medical devices’ heterogeneity, it is challenging to deploy and replicate software-based RA schemes that rely on strict hardware architecture configuration. Finally, the atomic execution of RA protocols that do not allow interruptability is a crucial challenge in healthcare considering the vital operation of medical devices.

## 8. Conclusions

In this work, we have presented the state-of-the-art of software-based Remote Attestation (RA) schemes, to analyze their applicability in the context of IoT security. We have analyzed and compared their design features and security capabilities. Currently, software-based RA approaches have been almost completely abandoned, mostly because of their limitations in detecting various cyber attacks and running on multi-hop networks. However, in this paper, we have discussed the opportunities of using these approaches in attesting some specific classes of IoT systems, namely legacy IoT devices and resource-constrained IoT devices (such as battery-free IoT devices and Fog based networks of IoT devices). Along with the opportunities, we have also highlighted some open research issues concerning software-based RA schemes. We believe that this study might help in reconsidering applications of software-based RA protocols in those scenarios where the hardware-based requirements of recent advanced RA schemes are not practically and realistically feasible.

## Figures and Tables

**Table 1:** Related work summary.

Work	Year	Focus	Survey	Software-Based RA
Armknecht et al. [27]	2013	Software-based framework	✗	✓
Steiner and Lupu [28]	2016	RA on Wireless Sensors networks	✓	✓
Abera et al. [32]	2016	RA overview on IoT	✓	✗
Maene et al. [30]	2018	Hardware-based RA	✓	✗
Gross and Sfyrakis [7]	2020	Hardware-based RA	✓	✗
Ambrosin et al. [29]	2020	Collective RA	✓	✗
This paper	2021	Software-based RA	✓	✓

**Table 2:** Overview of software-based RA methods.

Scheme	Method	Loose Time
Reflection [13]	Sequential memory access	✗
SWATT [4]	Random address access+Time constraint	✗
Pioneer [5]	Random address access+Time constraint	✗
PIV [36]	Memory filling+Fresh hash function	✓
Self-Modifying Code [37]	Fresh attestation program	✓
Proactive [38]	Memory filling+Memory mixing	✓
Distributed 1 [39]	Memory filling+Random address access	✓
Distributed 2 [39]	Memory filling+Random address access	✓
Memory Filling [40]	Memory filling+Random address access	✓
USAS [41]	Memory filling+Random address access	✓
Dataguard [42]	Dataguards variable boundaries	✓
Lightweight [43]	Memory filling+Single register attestation	✓
LRMA [44]	Random address access+Time constraint	✗

**Table 3:** Summary of software-based RA characteristics.

Scheme	Complexity	Challenge	Hash Function
Reflection [13],	O(n)	(0,M1)+(M2,L)	RIPEMD-160
SWATT [4]	O(nln(n))	nonce	RC4
Pioneer [5]	O(nln(n))	nonce	SHA-1
PIV [36]	O(n)	RHF	MQ polynomial
Self-Modifying Code [37]	O(nln(n))	Fresh attestation program	RC4
Proactive [38]	O(n)	$Id_{Verifier}$+nonce	SHA-1 or MD5
Distributed 1 [39]	$O(\frac{n\;ln(n)}{b})$	nonce	RC5 - CTR
Distributed 2 [39]	$O(m\times \frac{n\;ln(n)}{b})$	nonce	RC5 - CTR
Memory Filling [40]	$O(\frac{n\;ln(n)}{b})$	$Id_{Verifier}$ + $Id_{Prover}$ + nonce	RC5 - CTR
USAS [41]	O(nln(n))	nonce or response	RC4
Dataguard [42]	O(v)	nonce+e	SHA-1
Lightweight [43]	O(1)	Register value+Hash of local register value	✗
LRMA [44]	O(nln(n))	nonce	RC4

**Table 4:** Overview of software-based RA schemes w.r.t. required verifier knowledge.

Scheme	Mem. Cont.	Exact HW Config.	Network Delay	Used Mem.	Checksum
Reflection [13], Dataguard [42]	✓	✗	✗	✗	✗
SWATT [4], Pioneer [5], LRMA [44]	✓	✓	✓	✗	✗
PIV [36]	✓	✗	✗	✗	✗
Self-Modifying Code [37]	✗	✓	✗	✗	✗
Proactive [38], Distributed 1 [39], USAS [41]	✗	✗	✗	✓	✗
Distributed 2	✗	✗	✗	✗	✓
Memory Filling [40]	✗	✓	✗	✓	✗
Lightweight [43]	✗	✓	✗	✓	✗

**Table 5:** Detection capabilities w.r.t. Attestation Adversaries.

Scheme	Precomputation	Replay	Memory Copy	Compression	ROP
Reflection [13]	✓	✓	✗	✗	✗
SWATT [4]	✓	✓	✓	✗	✗
PIV [36]	✓	✓	✓	✗	✗
Self-Modifying Code	✓	✓	✓	✗	✗
Proactive [38]	✓	✓	✓	✗	✗
Distributed 1 [39]	✓	✓	✓	✗	✗
Distributed 2 [39]	✓	✓	✓	✗	✗
Memory Filling [40]	✓	✓	✓	✗	✗
USAS [41]	✓	✓	✓	✗	✗
Dataguard [42]	✓	✓	✓	✗	✗
Lightweight [43]	✓	✓	✓	✗	✗
LRMA [44]	✓	✓	✓	✗	✗
